# Supplementary material for: Radiation dermatitis in the hairless mouse model mimics human radiation dermatitis
Source: Sci Rep. 2024 Oct 22;14:24819. doi: 10.1038/s41598-024-76021-9 (PMC11496547; doi:10.1038/s41598-024-76021-9)
Supplement: Supplementary file 1 — Supplementary Material 1 [file 41598_2024_76021_MOESM1_ESM.docx]

**Supplementary Data**

Supplementary Table 1: Radiation-induced dermatitis grading scheme.

| **Grade** | **Description** |
| --- | --- |
| 0 | No change over baseline |
| 1 | Follicular, faint or dull erythema  Epilation  Patchy dry desquamation |
| 2 | Tender or bright erythema  Diffuse dry desquamation  Mild-moderate edema  Patchy non-dry desquamation |
| 3 | Diffuse non-dry glistening desquamation  Diffuse edema |
| 4 | Ulceration  Hemorrhage  Necrosis |
| 5 | Death due to dermatitis |

**Supplementary Figure 1.**


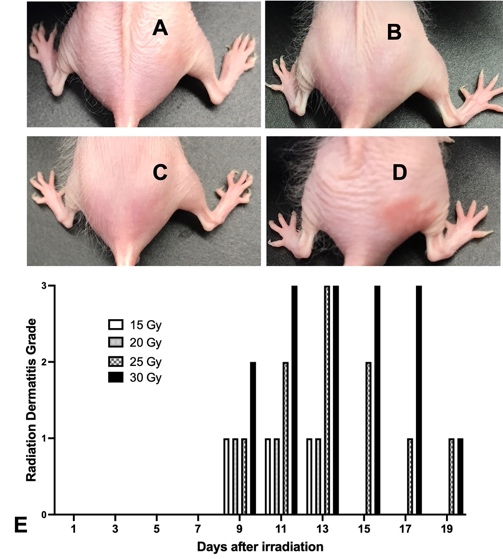


Radiation dermatitis in SKH-1 mice following pilot dose escalation study. SKH-1 mice (N=1 per dose) received one fraction of radiation at increasing doses directed to the skin over the right proximal hip. Photographs illustrating the radiation target site at peak toxicity following 15 Gy (A), 20 Gy (B), 25 Gy (C) and 30 Gy (D). Dermatitis grade was documented following treatment (E) to determine the radiation dose to be used in larger animal studies to produce persistent grade 3 dermatitis.

**Supplementary Figure 2.**


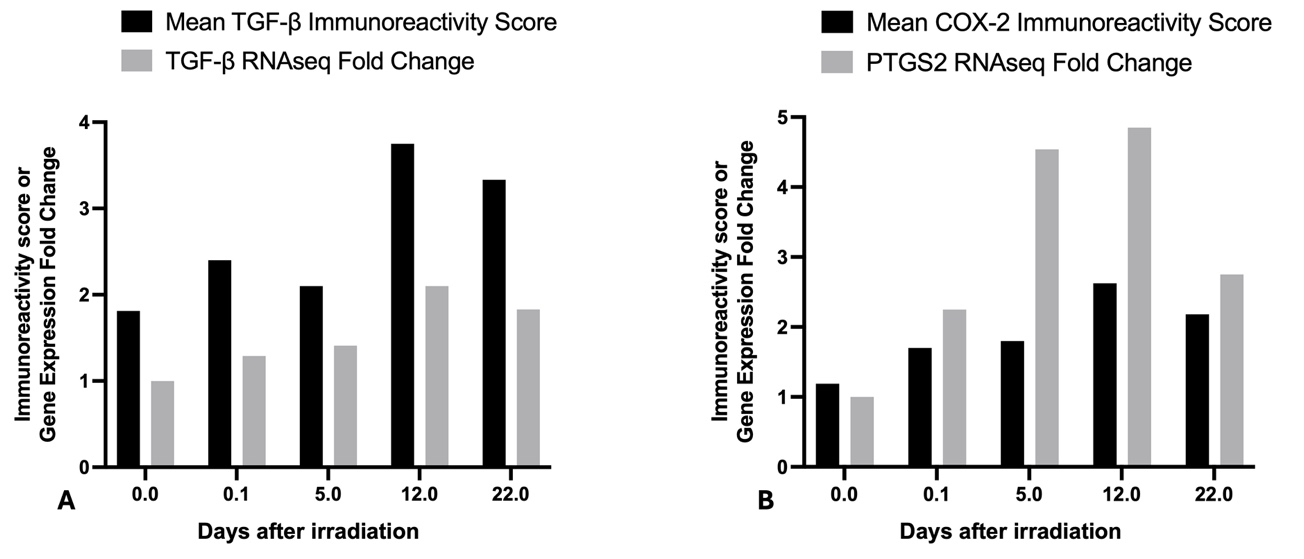


Pooled irradiated skin was acquired from mice (N=4) at each time point was assessed for gene expression changes in (A) TGF-β1 or (B) PTGS2, which codes for COX-2. Gene expression was determined using RNA sequencing. TGF-β1 and PTGS2 gene expression increased and peaked 12 days after irradiation, similar to mean TGF-β1 and COX-2 immunoreactivity scores, respectively, after irradiation. Immunoreactivity scores are shown as the mean of the dermal and epidermal immunoreactivity scores shown in Fig 4. TGF-β1 and PTGS2 gene expression data are presented as fold changes from pooled samples.
